# Motivation or demotivation of health workers providing maternal health services in rural areas in Vietnam: findings from a mixed-methods study

**DOI:** 10.1186/s12960-015-0092-5

**Published:** 2015-12-02

**Authors:** ᅟ Nguyen Thi Hoai Thu, Andrew Wilson, Fiona McDonald

**Affiliations:** Health Management Training Institute, Hanoi School of Public Health, Hanoi, Vietnam; Menzies Centre for Health Policy, University of Sydney, Sydney, Australia; Australian Centre for Health Law Research, Queensland University of Technology, Brisbane, Australia

**Keywords:** Motivation, Maternal health services, Vietnam, Human resources for health in low- and middle-income countries

## Abstract

**Background:**

Motivation is an important driver for health professionals to maintain their professional competencies, continue in the workforce and make a positive contribution to their workplace. While there is some research about the motivation of health workers in low- to middle-income countries, maternal morbidity and mortality remains high and this can be lowered by improving the quality of maternal health services and the training and maintenance of the skills of maternal health workers. This study examines the impact of motivation on maintenance of professional competence among maternal health workers in Vietnam using mixed methods.

**Methods:**

The study consisted of a survey using a self-administered questionnaire of 240 health workers in five districts across two Vietnamese provinces and in-depth interviews with 43 health workers and health managers at the commune, district and provincial level to explore external factors that influenced motivation. The questionnaire includes a 23-item motivation instrument based on the Kenyan health context, modified for Vietnamese language and culture.

**Results:**

The 240 responses represented an estimated 95% of the target sample. Multivariate analysis showed that three factors contributed to the motivation of health workers: access to training (*β* = −0.14, *P* = 0.03), ability to perform key tasks (*β* = 0.22, *P* = 0.001) and shift schedule (*β* = −0.13, *P* = 0.05). Motivation was higher in health workers self-identifying as competent or who were enabled to provide more maternal care services. Motivation was lower in those who worked more frequent night shifts and those who had received training in the last 12 months. The interviews identified that the latter was because they felt the training was irrelevant to them, and in some cases, they do not have the opportunity to practice their learnt skills. The qualitative data also showed other factors relating to service context and organisational management practices contributed to motivation.

**Conclusions:**

The study demonstrates the importance of understanding the motivations of health workers and the factors that contribute to this and may contribute to more effective management of the health workforce in low- and middle-income countries.

## Background

### Introduction

In developing countries, while improvements in maternal health (MH) have been remarkable, there is still significant room for improvement, particularly in disadvantaged regions. A number of reasons for the slow progress in achieving MH-related Millennium Development Goals have been identified in previous studies and reports, including the unavailability of MH workers, uneven distribution of the health workforce, and low motivation of health workers (HWs) [1]. International experience has demonstrated the critical roles that a HW could play in improving health outcomes but also in promoting human rights, accountability, innovation, political commitment and multi-stakeholder partnership [2, 3]. These issues remain relevant in the new era of development, moving forward with the newly determined Sustainability and Development Goals. Among the common problems and challenges affecting the development of human resources for health, low levels of the health workforce motivation is considered an important issue [3-6].

### Definition of motivation

Motivation is one of the most important factors affecting worker behaviour and performance. Motivation is described as something that energises individuals to take action and which is concerned with the choices the individual makes as part of his or her goal-oriented behaviour [7]. In the work context, motivation can be defined as “an individual’s degree of willingness to exert and maintain an effort towards attaining organisational goals” [8]. Motivation is a psychological process and a transactional process that results from the interactions between individuals and their work environment. It is a complex concept and is determined by factors at various levels [8, 9].

According to Frederick Herzberg’s well-known two-factor theory from 1959, two sets of factors, namely motivation and hygiene, influence employees’ working attitudes and level of performance [10]. Motivation factors are intrinsic factors, mainly related to the nature of the job, which increase employees’ job satisfaction. Hygiene factors are extrinsic factors that prevent employees’ dissatisfaction. Herzberg stated that a full supply of hygiene factors will not result in employees’ job satisfaction. In order to increase employees’ performance or productivity, motivation factors must be addressed [7].

Worker motivation is influenced by working conditions or hygiene factors [10, 11], including facility infrastructure and availability of resources; organisational support including supervision, training opportunities and professional promotion; and organisational structures and processes. Other contextual factors, including the characteristics of the population being served (e.g. client expectations), also influence worker motivation.

Motivation is considered an important but complex influence on the performance of HWs [3, 8, 12], and low motivation has a negative impact on the performance of individual HWs [13]. Health service delivery, service quality, efficiency and equity are all related to the willingness of HWs to mobilise their resources in performing their tasks [14]. Kanfer [15] identifies two aspects of the internal motivation process. The “will do” aspect involves the establishment of conformity between personal goals and the goals of the organisation (goal setting). The “can do” aspect involves the extent that individual resources are mobilised to achieve joint goals. This is dependent on workers’ perception of their competencies and the resources available in the working environment [16].

Worker motivation is an important but neglected facet of poor quality and low accessibility in healthcare, especially in developing countries [14]. Motivation among rural health workers, who are central in providing primary healthcare including maternal health services, is a neglected topic. More generally, the complex interaction of the social environment on health worker motivation and performance in low- and middle-income countries has been neglected in research [17]. This study aimed to identify contextual and organisational factors that influence the motivation of maternal health workers in rural districts in Vietnam. The term “client” used in this article refers to pregnant women who seek maternity services in a health facility.

### Conceptual framework

**Figure. 1:**
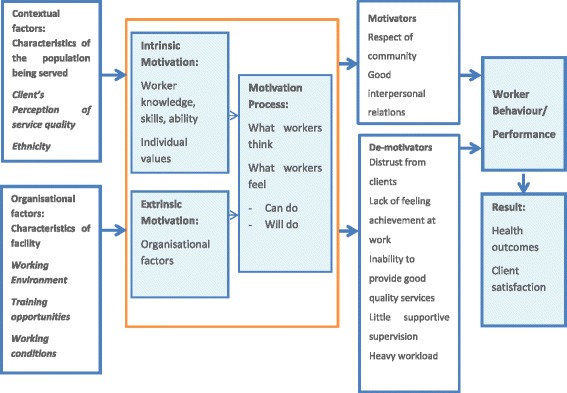
Conceptual framework for health worker motivation and demotivation

Motivation develops in HWs as a result of the interaction between individual, organisational and cultural determinants [8, 13, 18]. Some of these factors, such as traditional customs or a client’s perception of service quality, though they are more distal in nature, have a direct impact on HW motivation. Figure 1 describes how the various determinants are perceived by HWs in this study as motivating or demotivating factors.

Vietnam has 54 distinct ethnic groups, each with its own language, lifestyle and cultural heritage. In the following framework, the term “Ethnicity” is used here to refer to minority groups whose identity is based on common ancestral, social, cultural or national experiences.

## Methods

### Study locations and ethics

This study was conducted in five rural districts of two northern mountainous provinces of Vietnam, selected to represent different rural geographic and demographic characteristics. Participants included maternal health workers from c*ommune health centres* (CHC) and district hospitals. In the organisational structure of the Vietnamese healthcare system, the CHCs are designated to provide primary healthcare including MH services. The *district hospital* serves as the referral unit for all CHCs within a district.

Research ethics approval was obtained from the Queensland University of Technology research ethics committee. Permission for the research was granted from provincial health departments. Written consent was obtained from each participant.

*Study design*: A mixed-method approach was used to explore what motivates and demotivates maternal health workers in the five rural districts.

*The quantitative component* was a cross-sectional survey with the participation of all health workers (252) in the selected districts and communes, using a self-administered questionnaire. *The survey questionnaire* consisted of four sections namely the qualifications and experience of MH workers, their self-rated ability to perform the international standard essential obstetric care (EOCs) competencies [19] and relevant demographic information.

The questionnaire included a motivation scale developed by Mbindyo et al. [18] and modified following forward and backward translation to Vietnamese to allow for Vietnamese cultural and linguistic differences [20]. The original scale was a 23-item scale which consists of seven constructs, namely the following: general motivation, burnout, job satisfaction, intrinsic job satisfaction, organisational commitment, conscientiousness and timeliness and attendance [20]. A summated rating scale format is used, with five choices per item ranging from “strongly disagree” to “strongly agree”. Internal validity was confirmed using exploratory factor analysis. The loading of factors and extraction of factors were considered in relation to the original published factors. Based on the identified factors, reliability analysis was used to test the consistency of each construct of the motivation scale. Using principal component analysis with direct oblimin rotation, and applying Kaiser’s “Eigen values greater than one” rule, factors were retained that gave the most interpretable solution and best overall model fit. Items that did not load on any factors up to a level of 0.4 were eliminated. Multiple loading items were placed with best fitting factors conceptually. Eigenvalue and scree plot are two conventional criteria for determining the number of unrotated factors to be extracted [21]. Results of the factor analysis of the motivational questions showed six factors with eigenvalues higher than 1, and scree plot indicated that a six-factor model would be sufficient to represent the data [22, 23]. These six factors accounted for 61.2 % of the total variance. The six factors were defined as job satisfaction, workplace relation, timeliness and attendance, general motivation, burnout and conscientiousness with corresponding Cronbach’s alpha 0.87, 0.57, 0.51, 0.52, 0.74 and 0.79. Cronbach’s alpha of the whole scale was 0.77.

Data, after any necessary recoding, was double-entered using EpiData. Identified inconsistencies were checked against the original survey. The data was then transferred to IBM Statistical Package for the Social Sciences (SPSS) 19 for analysis [24]. Multivariate regression modelling was used to identify the independent effect of factors on total motivation scores using a hierarchical regression approach [25]. Total motivation score was calculated by sum of scores of 22 items in the motivation scale. The independent variables selected for entry in the model were “Gender”, “Received training course in the preceding 12 months”, “Ability to perform EOCs”, “Shift Schedule” and “Work Experience”. Detailed results are described in Table 1.

**Table 1:** Association of independent variables and total motivation scores

Variable	B	SE B	Β	*t*	*P*
Constant	86.52	4.07		21.26	<0.001
Gender	2.44	1.39	0.11	1.75	0.08
Male					
Female					
Received training in 12 months	−2.94	1.33	−0.14*	−2.21	0.03
Yes					
No					
Ability to perform EOCs	4.45	1.32	0.22**	3.37	0.001
0 = able to perform less than 75% of total number of EOCs					
1 = able to perform more than or equal to 75% of number of EOCs					
Number of years working in maternal healthcare	−0.09	.078	−0.08	−1.19	0.23
Below 5 years					
5 to 10 years					
More than 10 years					
Night shift schedule	−2.14	1.07	−0.13*	−2.0	0.05
1 = less than or equal to 4 days per month					
2 = 5–8 days per month					
3 = more than 8 days per month					

*N* = 224,* *p*<0.05, ** *p*<0.01 *R* ^2^ = 10 %

*The qualitative research* involved 43 in-depth interviews with participants from commune to central levels. Purposeful sampling was used to select key informants to include all health service levels, disciplines and geographic areas [26]. The approach utilised the “snow ball or chain” approach with the number of interviews determined by the point that responses to particular questions are saturated, that is, no new information is being added (Strauss and Corbin, 1998, cited by [27]). Semi-structured, open-ended interview guides were used to assist consistency in approach. Sources and methods were triangulated by interviewing HWs and their managers at the commune, district, provincial and central levels in order to assure the trustworthiness of data [28]. All transcripts were uploaded into NVivo 9.0 [29] and single-coded. The grounded theory approach [30] provided a way of synthesising data, developing concepts and also testing emergent concepts with additional fieldwork [26].

## Results

### Descriptive statistics

Of the 252 participants invited to participate in the survey, 240 returned the completed questionnaires (response rate 95 %). Table 2 describes the characteristics of participants.

**Table 2:** General characteristics of participants

Participant characteristics	Total	Percent
Gender (female)	173	72.1
Ethnic people	42	17.5
Age group		
20–29	31	13
30–49	181	75.74
50–60	28	11.6
Technical position		
Medical doctor	58	24.2
Assistant doctor^a^	84	35
Midwife	87	36.2
Others (nurses, technicians)	11	4.6
Years of working in maternal health area		
<5 years	56	23.3
5–10 years	70	29.2
>10 years	114	47.5
Qualifications		
Secondary degree^b^	166	69.2
University or equivalent degree	64	26.7
Postgraduate	10	4.2
Obstetrics expertise		
No	107	44.6
Yes	133	55.4
Night shift rostering		
<4 days/month	11	4.9
4–8 days/month	106	46.9
>8 days/month	109	48.2
Receive training in the last 12 months		
No	84	35.0
Yes	156	65.0

^a^Assistant doctors complete a 3-year training course in medicine and can prescribe

^b^Secondary degree requires 2 years training and is similar to vocational training

### Motivating factors for health workers providing maternal health services

#### Respect from the community

One of the important factors motivating HWs to stay in their job is the work itself. Being able to provide examinations and consultations for pregnant women, to assist their birth and to see newborn babies daily were considered a pleasure. “I work as a midwife, so every day I take care of women coming here for birthing and greet new babies, I love babies so I like my job” (District_Staff_2).

Many respondents expressed similar reasons for choosing to work in the health professions. Many became HWs due to the prestige associated with medical- and health-related work, and they were satisfied with their choice. Health workers considered that an important motivator was receiving respect from the local people who they served.

“The local people, if you are dedicated to them, they respect you and they are also interested in you, no trouble at all, in general the relation between us as health workers and people are good” (Commune_Manager_3).

#### Good interpersonal relations

In general, respondents acknowledged that they had good relationships with managers and colleagues. The social interaction among HWs was reported as being relatively friendly and close.

“This is one of our advantages. We are united and we collaborate well. We discuss with others and give comments for one another. Before implementing any task, we often reach the common agreement amongst HWs in CHC” (Commune level_Manager_3, Commune level_Staff_4).

Because each CHC has only a few staff, the relationships between them are considered to be close, like a family relationship. Many CHC workers (CHWs) agreed that information sharing and the work experience at CHCs is relatively favourable due to a good working atmosphere. CHWs also expressed their satisfaction with work-related feedback from their direct manager.

From the interviews with CHWs, “organisational commitment” was understood as having a stable job with sufficient income and living near family. For those participants from a rural background, being close to their home town and family was perceived as very important, and that appeared to be a central reason for their satisfaction with their current job and remaining in their positions. For young CHWs with a secondary degree or lower, it also seemed unlikely that they would find better opportunities in another place.

### Demotivating factors for health workers providing maternal health services

#### Distrust from clients

Negative perceptions of service quality by clients were reported to affect the utilisation of MH services and hence lower HW motivation. According to the CHWs in this study, delivery services in CHCs are seldom used due to women’s negative perceptions of the quality of services provided in CHCs.

“As they have not ever come to a CHC to give birth, they would not know if health workers working there could perform well. They perhaps do not trust in health worker competencies”. (Commune level_Manager_1)

Several reasons were reported why clients choose to fast track to higher level facilities for delivery services, including district hospitals being equipped with more modern medical equipment and with better infrastructure. Moreover, clients felt more trust in HWs at the district hospital than at the commune level because those HWs are seen as more specialised and professionally trained.

“…patients seek more a trustworthy address. That [the quality of service in CHC] is one of the patients’ concerns. Quite few of them bypass to higher levels. In fact, health staff should be specialised, and appropriately and professionally trained” (Commune level_Manager_3).

#### Perception of poor work achievements

The culture and beliefs of ethnic peoples strongly influence clients’ health-seeking behaviours and use of maternity services. The attendance level of ethnic women at some CHCs for MH services was reported to be low resulting in CHWs being unable to accomplish their tasks in MH areas. For example, CHWs could not achieve the set targets for the proportion of women having more than three antenatal care episodes or women birthing with the assistance of skilled birth attendants. This was considered by many respondents to lower the morale and motivation of HWs.

Many ethnic communities do not see any advantage in delivering at a public health facility, with women preferring the comfort and support of family and traditional birth attendants [31]. CHWs explained why ethnic women do not come to use maternity services in CHCs.

“Not many women come for antenatal care (ANC). They might be shy, or they might think it is not necessary, or they just do not know that it is necessary. So only a few come.....To be honest, with this qualification, I will not have chance to use all the knowledge I learnt. In the long run, my competencies might be eroded. I need to have opportunities to practice” (Commune level_Manager_1).

Respondents reported that due to patients’ culture, the decision to seek MH services might be influenced by the family hierarchy where decision-making involves more members of the family, notably the husband.

Moreover, language barriers may also impact on attendance. CHWs in mountainous areas reported difficulty in communicating with ethnic pregnant women since most women do not speak the Kinh language (the primary language in Vietnam). Husbands or local people translated between HWs and women. This was explicitly described as one obstacle for CHWs implementing the health communication and education programmes for pregnant women.

“So hard, because it relates to population literacy. Most pregnant women do not speak Kinh language, they just speak ethnic language when coming to the CHC”. (Commune level_Manager_1)

Health workers at CHCs, especially in scattered mountainous areas, report that they often feel that they are poor performers, when the service target (at least 70–80 % of pregnant women have prenatal checks) is continually not achieved.

#### Inability to provide good quality services

Infrastructure constraints were reported to affect health workers’ ability to serve patients, including unavailability of utilities, shortages of drug supplies and old buildings. Respondents in one province reported that while electricity was available at almost all CHCs clean water was infrequently supplied. In some CHCs, HWs had to fetch clean water by hand from the foot of a hill far from the CHC for cooking. “There was also not enough clean water for bathing and washing, so it was difficult to handle a delivery” (Commune level_Staff_1). Lack of clean water in the mountainous areas was perceived as having reduced the utilisation of MH services. Although clean water was a pressing and urgent issue for CHCs in one district, the solution taken by local authorities to address it appeared largely ineffective.

“We reported many times, and every year the District People’s Committee provides plastic or rubbery pipe to bring water to CHC. The pipe is normally an average length of 20–30 meters but there are some places that need a couple of hundred of meter pipe to go across several hills. The plastic pipe over the hills might be ruined due to buffalos and cows left unbridled or during harvesting time”. (District level_Manager_1)

The shortage of service rooms was reported as leading to room sharing in most CHCs resulting in concerns about cross-contamination. Not many CHCs had the required six separate rooms or the minimum four rooms for reproductive health (RH) care as defined in the National Guidelines on RH [32]. Therefore, many RH services had to be performed in one room.

“The delivery attendance, gynaecology examination and family planning services have shared one room for a long time and it caused cross infection from gynaecological patients to women who came to birth or use family planning services. Now it needs to separate these rooms”. (Commune level_Manager_2, District level_Manager_14)

This fact has caused concern for CHWs about the safety of clients who come to use services in CHCs.

A lack of medical equipment and drugs was considered to impede CHWs’ ability to perform EOCs. The availability of magnesium sulphate is one typical example. Respondents acknowledged in interviews that almost all CHCs at the study locations did not have this drug, even though it is considered one of the essential drugs that should be available at any time in appropriate quantities.

#### Training opportunities were not effective in improving knowledge and skills

Pre-service training and in-service training play a critical part in ensuring workers have appropriate knowledge to perform their tasks, as well as to maintain their competencies over time. However, despite this broadly accepted principle, training opportunities were perceived to not be effective in motivating health staff working in rural areas.

Respondents reported finding it hard to balance training and working time since most training courses were organised in the last quarter of the year, the period when CHWs are busier than usual with reports and completing other tasks. Due to budget unpredictability, the organisation of training courses was reported as incompatible with HW demand in terms of timing. Programming training in this way may fail to improve HW knowledge and skills since the participation rate may be low. Such disorganisation means that that training is a less effective health resource management (HRM) tool for maintaining motivation.

In the study provinces, although HWs at the commune and district levels had recently experienced training associated with vertical programmes and other foreign aid projects for basic MH knowledge, respondents claimed that training content sometimes was not relevant to what they needed in practice. One respondent shared her experience in attending a training course:

“…content of training course is very little and not intensive, because these courses are only one day or 2-3 days. Participants only attend the lecture, not practice. If trainee really pays attention, he can acquire some knowledge but if he does not, he gets nothing” (Commune level_Manager_2).

The content of current training courses had been developed for HWs generally but requires adjustment to the local conditions to ensure HWs can apply what they learn at their work place. The training method was perceived as inappropriate and ineffective in terms of improving HW competencies, motivation and performance. HWs stated that they actually needed to practice in real situations or needed hands-on training in order to learn clinical skills that make them confident to perform as indicated by this response: “I think it would be much better if we can provide HWs with training by practice. Short courses from 3-5 days only give them theory, learn on imitation, no practice” (Provincial level_Administrator_3).

Respondents also stated that the evaluation of the training outcomes was either not undertaken or inadequate. Evaluations were confined to testing the knowledge of participants after the training, rather than checking that participants could perform the new skills confidently. In general, HWs at both commune and district levels expressed their dissatisfaction with the current training opportunities provided to them.

These qualitative results are in line with quantitative results presented in Table 1. This table shows the result of multivariate regression modelling which was used to identify the independent effect of factors on total motivation scores. The selected independent variables included gender, received training in preceding 12 months, ability to perform EOCs (the cut-off point is 75 % of EOCs), number of years working in the MH area and night shift schedule. The basic information of these selected variables was detailed in Table 2. The factor “Received training in the preceding 12 months” was weakly negatively associated with total motivation scores (*β* = −0.14, *P* = 0.03).

#### Night shift and extra workload = heavy workload

Table 1 shows that night shift schedule was also associated with motivation scores, such that those participants who had more frequent shift schedules (five to eight nights or more per month) were likely to have lower motivation scores (*β* = −0.13, *P* = 0.05). Most participants from CHCs and the obstetric department of the district hospital (DH) work night shifts, and many of them had more than eight nights per month. The high frequency of night shifts was reported as an issue by HWs at both the district and commune levels. Working night shifts was seen as more challenging as many district hospitals assign only one medical doctor in charge of all after hours cover, due to the lack of medical doctors.

“In one night shift at our hospital there is only one medical doctor but he has to handle all the things, including the process to admit patients at night, doing examination and treatment, or handle emergency cases. So it’s very hard for us to manage things during night shift.” (District level_Staff_1).

Additional concerns raised by respondents included that the frequent night duty was coupled with unsatisfactory allowances and especially affected female HWs since they are expected to take care of their families. “I do not like to have frequent night shifts because I have to leave my children at home. We are women, you know. We do not want to work at night, while the allowance is very low. My children are still small that need being cared and supervised.” (Commune level_Staff_2).

Apart from the high frequency of night shift rostering, a heavier workload caused by multiple roles was reported as an issue. Many district hospitals reported difficulty in assigning tasks for their HWs due to staff shortages, particularly during night shifts. For example, one manager could be assigned to two positions at the same time, such as the head of the obstetric department and vice head of the planning department. The workload of both departments was very heavy due to HR shortages. One manager illustrated the impact of staff shortages on her daily work; she noted:

“I am the head of obstetric department but still have to sit in consulting rooms because we lack HWs, so I feel very tired. Working hard and feeling tired, but sometimes I also have to replace the staff in ultrasound room when he is off. In general I have to work in different departments”. (District level_Manager_11)

This problem was not restricted to district-level HWs. CHWs also have to take responsibility for many roles. In practice, CHCs are designed to implement a wide range of vertical programmes and services. National vertical programmes reportedly placed additional demands on HWs such as writing reports, filling forms and attending meetings.

#### Little supportive supervision

Poor supervision was viewed as demotivating by some respondents. Supervision was conducted monthly or quarterly depending on the schedule of districts. In general, the respondents working at the CHCs felt that supervision they received from the district level was helpful when it provided information and instructions and identified areas for improvement. However, the quality of supervision did not fully meet the expectations of CHWs because supervisors lacked supervision skills. This in turn was seen as demotivating for CHWs. On questioning, the supervision turned out to be largely checking compliance with processes for CHCs’ reports and records as required in the current reporting system. The amount of time supervisors spent at the CHCs, reflecting the lengthy travel requirements in remote and mountainous areas, was also seen as unsatisfactory.

“To be honest, I think supervision is not efficient. Because they just come to check our reports, to see if we can do some services or not and remind us that we need to do. The support for technical aspect is not efficient. When we ask about how to deal with some specific cases, we do not get the answer”. (Commune level_Manager_2)

## Discussion

The data presented suggests that, to better maintain or increase motivation among MHWs in rural areas, it may be useful for managers to examine the motivators and demotivators identified in this study. Identified motivators include the following: the respect of the community and good interpersonal relations. Demotivators include the following: distrust from clients, a lack of a feeling of achievement at work, an inability to provide good quality services, little supportive supervision and heavy workloads. Although the participants in this research were maternal health workers, results can be extrapolated to health workers in the wider health system.

It is broadly accepted that health workers with a rural background tend to be more willing to work in rural areas and should therefore be actively encouraged to choose a rural post through targeted recruitment programmes [33]. Many of them chose to join the health professions because they wanted to help people and because the health professions are highly respected [34]. Additionally, good working relationships among colleagues and between staff and managers also enhance HW motivation. So, what demotivates them? From this study of health workers in a rural area of Vietnam, we came to two major conclusions that can inform HRM policy and practice and address gaps in the literature.

First, the cultural context and characteristics of the population being served were considered to be one of key contributors to HW motivation and performance [3, 8]. According to Kak and Burkhalter [35], cultural and social factors encompass community expectations, peer pressure, patient expectations and social values. An increasing number of studies have focused on cultural and social contexts, including the interaction between HWs and clients, recognition of supervisors or support of family and community [17, 36], employer or client/community recognition [37] and respect from the community [9, 17, 34]; though little has been reported on the influence of these factors on health worker motivation.

In this study, we found that the health services at many communes and districts are underutilised, though reasons are varied. In one province, local people have high expectations of quality and do not trust the capacity of CHCs, so they often bypass CHCs to attend district or provincial hospitals. In the second province, where most of the population are ethnic people with special customs and beliefs, they do not use maternity services. Regardless of the reasons, low utilisation of services, in turn, affects health staff motivation in at least two ways. First, low utilisation results in fewer opportunities for practice to maintain the skills of staff in these areas. It also was seen to impact on career aspirations. A plausible explanation was provided by respondents that HWs do not see a career pathway (a motivator in the two-factor theory [7]) in long-term employment at CHCs, where there are not many women coming to seek maternity services. Qualified health workers, therefore, tend to move to district health facilities to work, such as a district hospital where they have patients and they can practice what they have studied. Second, low utilisation conflicts with external performance requirements and therefore creates the potential for the service to be seen to be unsuccessful. Many respondents from Lao Cai province suggested that achieving the CHC’s targets for maternity services was an “impossible mission”. This perception could impact on staff motivation defined as “an individual’s degree of willingness to exert and maintain an effort towards organisational goals” [8]. Work motivation according to Franco and Bennett [16] exists when there is alignment between individual and organisational goals, when achievement of organisational goals is associated with personally desired outcomes, such as a sense of achievement or personal gain. In the study provinces, working at a CHC, especially in mountainous areas, health staff often reporting thinking of themselves as poor performers; consequently, they lacked a feeling of achievement. This result resonates with other published work in low- and middle-income countries [18, 33, 38].

The second group of factors identified were non-wage job attributes, such as training opportunities, career development prospects and living and working conditions, and these play a role in what job health workers choose [33] and influence their motivation [12, 18, 39].

Training opportunities are usually associated with higher motivation scores [40], are broadly considered a motivating factor [9, 28] and are positively associated with satisfaction [41]. Contrary to previous studies, our results showed a negative relationship between access to training in the preceding 12 months and total motivation scores. The reasons for this include HWs’ perception of training opportunities as inappropriate in terms of scheduling and the relevance and usefulness of the content and training methods. A similar situation has been reported from African settings where governments identified a lack of specialist training for their MH workers and there were no standards for clinical training courses [42]. In-service training is an essential HRM tool to improve or maintain skills and improve HW motivation, and in turn, this improves HW performance and quality of health services. In order to have the most likelihood of a positive impact, planning and delivery of training needs to include formal and locally based needs assessment (e.g. the relevance of training topics and contents, time to organise training courses, material adaptation) and attention to the appropriateness of training methods [43].

A recent report from the Vietnamese Ministry of Health shows that the investment of resources in infrastructure and medical equipment and funds for regular operation of preventive medicine, including reproductive health, are inadequate and do not keep up with demand [44]. Indeed, the probability of accomplishing EOCs very much depends on the resources provided for health facilities at the grassroots level. The inadequacy of health facility buildings and the lack of essential health technologies at CHCs (such as magnesium sulphate for treatment of severe pre-eclampsia and eclampsia) will result in incomplete implementation of the best technical practices as described in the National Guidelines. In such cases, the motivation of HWs was challenged by their inability to meet the quality standards, as well as client expectations [18]. Deficits in any components related to working conditions will not only affect the quality of health services but also decrease staff job satisfaction [45] and motivation [46].

Heavy workload is an important component of the working environment caused by inadequate staffing [47]. One aspect of workload, being assigned multiple roles, was reported by many respondents. HWs became overwhelmed by a broad range of tasks, such as writing reports, filling forms and attending meetings, with limited time for rest, and this was seen to negatively affect the quality of services and their motivation. The second aspect of workload was the frequency of night shift rostering. The results suggested that night shift duties in CHCs might not be as heavy as in district hospitals since there are fewer patients using services [34]. However, concerns raised by respondents at both levels included that frequent night duty was coupled with unsatisfactory allowances and negative impacts on the families of HWs, especially women. These results are consistent with previous research showing that frequent shift work is negatively correlated with HW motivation and satisfaction [36] and that a heavier workload is considered to demotivate HWs [34].

Supervision can be considered to contribute to “can do” and “will do” aspects of HW motivation [13]. The findings of this study identified that CHWs were dissatisfied with the supervision they received, claiming that it did not help to improve their performance. It is generally recognised that if supervision is implemented correctly, “it could become a mechanism for providing professional development, improving HW job satisfaction, and increasing motivation” [12]. However, ineffective supervision contributes to low staff morale, productivity and performance [39, 48].

## Conclusion

The most disadvantaged part of the health workforce are those working in difficult mountainous and rural environments with limited resources coupled with little opportunity to practice to maintain and develop professional competencies and with poor supervision. Highly trained professionals working in these areas see their knowledge and skills as being eroded, and this affects their motivation. Health system improvements should target areas that will most enhance motivation and retention of rural health workers. The results from this study suggest that effort should focus on providing healthcare workers with the appropriate medical equipment, drug supplies and infrastructure they need to deliver high-quality care. In addition to being necessary for health workers to perform their job, good human resource management needs to allow for adequate opportunity for HWs to practice, improve their knowledge and maintain their skills. Provision of in-service training should take into account the relevance of the training to the needs of HWs and adopt a practical approach that essentially accelerates the development of the task-relevant skills and competencies of HWs, while the supervision mechanisms should be reviewed so that it contributes to boosting the quality of care and worker motivation.
